# A survey of methodologies on causal inference methods in meta-analyses of randomized controlled trials

**DOI:** 10.1186/s13643-021-01726-1

**Published:** 2021-06-09

**Authors:** Georgios Markozannes, Georgia Vourli, Evangelia Ntzani

**Affiliations:** 1 Department of Hygiene and Epidemiology, University of Ioannina School of Medicine, Ioannina, Greece; 2 Deptartment of Hygiene, Epidemiology and Medical Statistics, Medical School, National and Kapodistrian University of Athens, Athens, Greece; 3 Center for Evidence Synthesis in Health, Department of Health Services, Policy and Practice, School of Public Health, Brown University, Providence, RI USA; 4 Institute of Biosciences, University Research Center of Ioannina, University of Ioannina, Ioannina, Greece

## Abstract

**Background:**

Meta-analyses of randomized controlled trials (RCTs) have been considered as the highest level of evidence in the pyramid of the evidence-based medicine. However, the causal interpretation of such results is seldom studied.

**Methods:**

We systematically searched for methodologies pertaining to the implementation of a causally explicit framework for meta-analysis of randomized controlled trials and discussed the interpretation and scientific relevance of such causal estimands. We performed a systematic search in four databases to identify relevant methodologies, supplemented with hand-search. We included methodologies that described causality under counterfactuals and potential outcomes framework.

**Results:**

We only identified three efforts explicitly describing a causal framework on meta-analysis of RCTs. Two approaches required individual participant data, while for the last one, only summary data were required. All three approaches presented a sufficient framework under which a meta-analytical estimate is identifiable and estimable. However, several conceptual limitations remain, mainly in regard to the data generation process under which the selected RCTs rise.

**Conclusions:**

We undertook a review of methodologies on causal inference methods in meta-analyses. Although all identified methodologies provide valid causal estimates, there are limitations in the assumptions regarding the data generation process and sampling of the potential RCTs to be included in the meta-analysis which pose challenges to the interpretation and scientific relevance of the identified causal effects. Despite both causal inference and meta-analysis being extensively studied in the literature, limited effort exists of combining those two frameworks.

## Background

Evidence-based medicine is an approach to medical practice defined as conscientious, explicit, and judicious use of current best evidence in making decisions about the care of individual patients in the light of their personal values and beliefs [1]. On a clinical research level and for pertinent research questions, randomized controlled trials (RCTs) undoubtedly offer the highest level of evidence compared to other study designs. An RCT’s primary objective is the minimization of biases, such as selection or allocation biases, by randomizing participants into the study groups in an unbiased fashion. If randomization is successful, the characteristics of the groups are expected to be equally allocated, therefore making the groups exchangeable. Under a complete protocol adherence and no loss-to-follow-up, this property of the RCTs essentially justifies the interpretation of the studied associations as the best available proxy of causal relationships [2]. When a randomized design is not feasible, data from observational study designs can be used to emulate a randomized experiment based on causal inference approach to obtain a valid causal estimate [3, 4]. Under a causal inference framework, the goal is to identify and compute that effect estimate that has a causally relevant interpretation on the population the trial samples from.

Meta-analysis is a quantitative procedure of assessing and combining data from multiple studies. By combining evidence from RCTs using meta-analytical approaches, one can potentially achieve higher levels of evidence. One caveat of this approach is that while each study’s estimate can potentially have a causal interpretation, their aggregation may lose this capacity, mainly due to differences on inherent study characteristics including (but not restricted to) differences in populations, in treatments and/or on the definition of outcome across studies.

In the present effort, we aim to identify and review methodologies relevant to implementation of a causally explicit framework for meta-analysis and discuss the interpretation and scientific relevance of that causal estimand. Therefore, in the first part, we focus on the published methodologies that address the identification and estimation of causal effects derived from meta-analyses of RCTs along with the underlying assumptions. In the second part, we go one step back in order to discuss the plausibility of the assumptions and issues concerning on the generalizability and scientific relevance of the derived estimands.

## Methods

### Definitions

We briefly present the causal inference framework known as the Rubin causal model [3, 5]. Let *Y* denote the outcome of interest and let *T* denote the treatment in a randomized trial. For simplicity, let the treatment take the values 1 for treated and 0 for untreated. Then, *Y*_*i*_^*1*^ denotes the potential outcome (counterfactual) of unit *i* under treatment, while *Y*_*i*_^*0*^ the potential outcome of unit *i* under no treatment. The quantity *Y*_*i*_^*1*^ *– Y*_*i*_^*0*^ denotes the difference in potential outcomes for unit *i*, the individual treatment effect (ITE). The quantities *Y*_*i*_^*1*^ and *Y*_*i*_^*0*^ can never be simultaneously observed for the same individual. The fact that for each *i* one of the *Y*_*i*_^*1*^ and *Y*_*i*_^*0*^ is always missing prohibits us from estimating the ITE. This problem is known as the “fundamental problem of causal inference” [5]. While ITEs are never observable one can estimate the average treatment effect (ATE). Under the assumptions presented in Table 1, one can use the observed quantity E[*Y*_*i*_^*1*^ | *T* = 1] – E[*Y*_*i*_^*0*^ |*T* = 0] as an estimator of the ATE = E[*Y*_*i*_^*1*^] – E[*Y*_*i*_^*0*^].

**Table 1:** Core assumptions for identifiability in causal inference

**Stable unit treatment value assumption (SUTVA):** The stable unit treatment value assumption states that there is no interference among units, that is, the treatment status of a unit does not affect the potential outcomes of other units and it also requires that there is only a single version of the treatment (no hidden variations in treatment; no multiple versions of treatment). Possible violations of the SUTVA include settings where units interact (e.g., schools, group interventions) or different treatment dosages exist or different modes of administration operate which can affect the potential outcomes.	
**Consistency:** An individual’s potential outcome under the observed exposure history is precisely the observed outcome: If *T* = *t*, then *Y*_*i*_^*t*^ = *Y*_*i*_	
**Positivity:** The probability of being assigned to each of the treatment levels is greater than zero for each level of a variable X: Pr(*T* = *t*|*X* = *x*) > 0	
**Assignment mechanism–ignorability:** Also known as exchangeability, or unconfoundedness, this assumption states that treatment assignment is independent of the potential outcomes; this roughly translates to no unmeasured confounders and no informative censoring. Ignorability can be either unconditional or conditional.	
• ***Unconditional ignorability:*** In RCTs, where the treatment is randomly assigned, the potential outcomes will be independent of the treatment assignment. Formally, this is defined as (*Y*_*i*_^*1*^,*Y*_*i*_^*0*^) ⊥ *T.* This stems from the main property of randomization, i.e., any measured or unmeasured confounder will be equally distributed across groups.	
• ***Conditional ignorability***: In non-randomized settings, confounders are not bound to be equally distributed across treatment groups, and thus unconditional ignorability cannot hold. However, given a set of covariates ***X*** and assuming that no unmeasured confounder exist, conditional ignorability can be defined as (*Y*_*i*_^*1*^,*Y*_*i*_^*0*^) ⊥ *T* | ***X***_*i*_.	

If these assumptions hold, one can make valid causal inferences. The positivity and ignorability assumptions are often considered together and are referenced as the *strong ignorability assumption*.

### Search algorithm, inclusion, and exclusion criteria

We performed a systematic search in four databases (Wed of Science, PubMed, Arxiv, and Google Scholar) using the search algorithm: “(causal* OR causat*) AND (meta–analysis OR metaanalysis OR “meta analysis” OR multilevel OR multilevel OR “multi level” OR hierarchical OR meta–synthesis OR “meta synthesis” OR metasynthesis)” from inception to April 2020 to identify relevant methodologies. The search was supplemented with manual searches and reference screening of all relevant studies. We focused on identifying studies that presented a causally explicit framework under a meta-analysis model for randomized controlled trials. We included methodologies that described causality under counterfactuals and potential outcomes frameworks. Studies that claim causality using only the Bradford–Hill criteria were excluded. We also excluded studies on Granger causality, which is more pertinent to prediction than causation. Studies that describe causal inference approaches which are not pertinent to evidence synthesis and application studies were excluded.

## Results

### Systematic methodology review

**Fig. 1:**
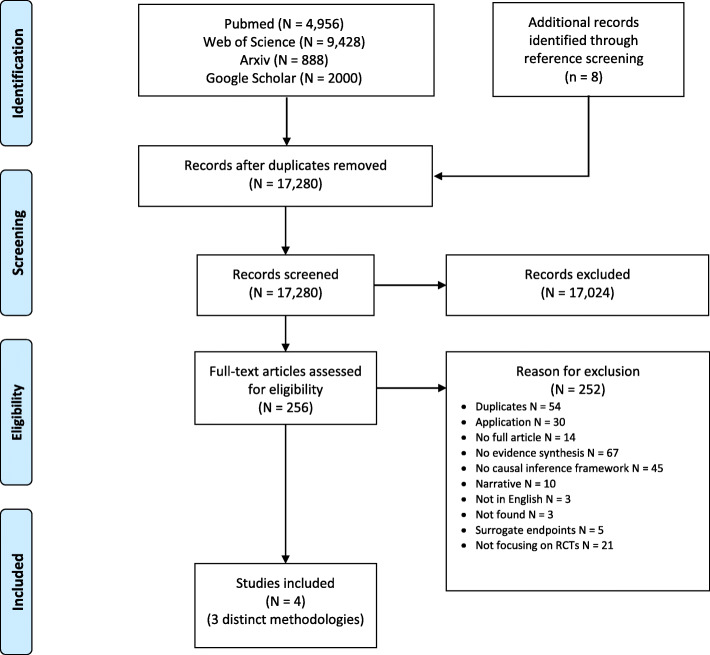
Flowchart

The search algorithm yielded a total of 17,280 titles. After initial screening, a total of 256 articles were screened in full-text for eligibility. Finally, only three distinct methodologies from four publications [6–9] describing for a causal inference framework in a meta-analysis setting were included in this review (Fig. 1). Two methodologies were based on a meta-analysis setting using individual participant data and the last one on a network meta-analysis setting using summary data. Below, we provide a brief description of those methodologies.

### Causal inference for meta-analysis using IPD data from independent RCTs

Sobel et al. [6] described a framework where causal estimates can be derived from a meta-analysis of RCTs when individual participant data (IPD) are available. The authors focus their work on identifying and accounting for possible sources of heterogeneity across trials. They restrict their focus on four possible sources of heterogeneity across trials: response inconsistency, non-equivalent treatments, non-ignorable treatment assignment, and variability in the composition of units in different studies or settings. Identifiability conditions taken into account in Sobel et al. approach are presented in Table 2.

**Table 2:** Sobel et al. identifiability conditions

***A1.*** *Extended stable unit treatment value assumption (eSUTVA): For all possible assignments t and allocations s, Y*_*i*_(*s; t*) = *Y*_*i*_(*s*_*i*_*; t*_*i*_) *≡ Y*_*i*_(*s; t*)	
**A2.** Study sampling assumption: For all subjects *i* in study *s*, *s* = 1,…, m, the random vectors *Y*_*i*_; **X**_i_ | *S*_*i*_ = *s*; *T*_*i*_ = *t* are independent and identically distributed *Y*; **X |** *S* = *s*; *T* = *t*	
**A3a.** Strong response consistency assumption for treatment t: For all *s*; *s’* and subjects *i*, *Y*_*i*_(*s; t*) = *Y*_*i*_(*s’; t*)	
**A3b.** Weak response consistency assumption for treatment *t*: For all *s*, *s’* and **X**: F (*y*(*s; t*) | *S* = *s’*; **X** = **x**) = F (*y*(*s’; t*) | *S* = *s’*; **X** = **x**)	
**A4.** Weak consistency of effects of treatment *t* versus *t’*: For all *s*, *s’* and ***X***, the causal estimands: H (F (y(*s; t*) | *S* = *s’*; **X** = **x**); F (y(*s; t’*) | *S* = *s’*; **X** = **x**)) = H (F (y(*s’; t*) | *S* = *s’*; **X** = **x**); F (y(*s’; t’*) | *S* = *s’*; **X** = **x**))	
**A5a.** Strong equivalence of treatments *t*_*1*_ and *t*_*2*_ in study *s*: For all *i*: *Y*_*i*_(*s; t*_*1*_) = *Y*_*i*_(*s; t*_*2*_)	
**A5b.** Weak equivalence of treatments *t*_*1*_ and *t*_*2*_ in study *s*: F (*y*(*s; t*_*1*_) | *S* = *s*; **X** = **x**) = F (*y*(*s; t*_*2*_) | *S* = *s*; **X** = **x**)	
**A6.** Unconfounded treatment assignment given observed covariates: for every *s*, and treatment *t* ∈ ***T***_*s*_, F (*y*(*s; t*) | *T* = *t*; *S* = *s*; **X**_1_ = **x**_1_) = F (*y*(*s; t*) | *S* = *s*; **X**_1_ = **x**_1_)	
**A7.** Unconfounded study selection, given observed covariates: For all studies *s*, *s’* and treatments *t*, F (*y*(*s; t*) | *S* = *s*; **X**_2_ = **x**_2_) = F (*y*(*s; t*) | S = *s’*; **X**_2_ = **x**_2_)	

#### Notation

Let *t* denote the treatment with *t* ∈ ***T*** = (1,…, L) where ***T*** is a finite set of treatments. Then, ***T***_*s*_ denotes the set of treatments in study s. Let *s* denote the trial with *s* ∈ ***S*** = (1,…, m) where ***S*** is a finite set of trials. Let **X**_i_ be a set of observed covariates, including both subject-level and trial-level covariates for subject *i*. *Y*_*i*_ ≡ *Y*_*i*_(*s*_*i*_*; t*_*i*_) is the observed outcome for subject *i* in study *s* under treatment *t*.

The authors extend the potential outcome framework to multiple studies by considering the potential outcomes a subject would have had should he/she participated in a different trial. Let ***t*** = (*t*_*1*_*,…, t*_*n*_) and ***s*** = (*s*_*1*_*,…,s*_*n*_), where *t*_*i*_ ∈ ***T***, *s*_*i*_ ∈ ***S***, *i* = 1,…,n, and let *Y*_*i*_(***s****;* ***t***) denote the response subject *i* would have under the allocation ***s*** to studies and assignment ***t*** to treatments.

Assumption A1 is the extension of SUTVA under multiple trials and treatments. A2 denotes that each trial includes a random sample from its respective population. A3a and A3b state that the effect of a treatment is the same across studies unconditionally or conditional on covariates. A4 further weakens these assumptions stating that the relative effect of treatment *t* versus treatment *t’* is the same across studies, unconditionally or conditional on covariates. A5a and A5b denote that although different versions of the treatment may exist, their effect on the potential outcomes is equivalent. This allows several treatments to be grouped together. A6 is the classic unconfoundedness assumption. If all studies are randomized trials, this assumption is expected to hold unconditionally and conditional on ***X***_***1***_. Based on A7, the authors explicitly acknowledge that different trials may sample their subjects from different populations, but assume that given a set of covariates ***X***_***2***_, subject assignment into trials is unconfounded. The authors comment that some of these assumptions are untestable by themselves, but if a number of those are assumed to hold, one can then test the plausibility of them holding given the other assumptions holding. Overall, this framework does not use a complex analytical approach, rather is being based on the plausibility of the aforementioned assumptions to hold and by a correct model specification using study level covariates and possibly treatment, study, and covariates interactions. The authors applied a standard Cox model to estimate the causal effect which they justified based on A7.

Dahabreh et al. [7, 8] proposed a causal inference framework under which meta-analysis estimates are causally interpretable and transportable ATEs to a target population. This approach requires IPD from the randomized trials along with baseline covariate data from a random sample from the target population, in order to account for differences in distributions. They provide a set of assumptions for identifiability conditions and also propose an estimand that takes into account the distributional differences between trials and target population (Table 3). This framework assumes that the observed data are obtained by random sampling from an infinite superpopulation of individuals which is stratified by study *S*. Authors denote this sampling method as a “biased” sampling since the proportion of the sampled population is not expected to be equal with the superpopulation due to convenience sampling in the majority of the RCTs. This framework assumes complete adherence to the trial protocol and no loss-to-follow-up, leading to the intention-to-treat effect being equal to the per-protocol effect.

**Table 3:** Dahabreh et al. identifiability conditions when pooling trials

***B1****. Consistency of potential outcomes: If T*_*i*_ = *t; then Y*_*i*_^*t*^ = *Y*_*i*_*, for every individual i in the target population or the populations underlying the trials in S*	
***B2****. Conditional exchangeability over treatment assignment T:* *E*[*Y*^*t*^ ∣ **X** = **x**; *S* = *s; T* = *t*] = *E*[*Y*^*t*^ ∣ **X** = **x**; *S* = *s*], *for every trial s* ∈ **S***, each treatment t* ∈ **T***, and every*** x ***with f*(**x;** *S* = *s*) > *0*	
***B3****. Positivity of the treatment assignment probability in the trials:* *For every treatment t* ∈ *T, Pr*[*T* = *t* ∣ **X** = **x**; *S* = *s*] > *0 for every trial s* ∈ **S ***and every* **x ***with f* (*x;* *S* = *s*) > *0*	
***B4****. Conditional exchangeability in measure between the trial and the target population: For every pair of treatments t and t′ in* **T***, E*[*Y*^*t*^ – *Y*^*t* ′^∣ **X** = **x**; *S* = *0*] = *E*[*Y*^*t*^ – *Y*^*t* ′^∣ **X** = **x;** *S* = *s*] *for every trial s* ∈ **S ***and every*** x ** *with f* (**x**; *S* = *0*) > *0*	
***B5****. Positivity of the probability of participation in the trials: Pr*[*S* = *s* ∣ **X** = **x**] > *0 for every s* ∈ **S ***and every* **x** *with f* (**x**; *S* = *0*) > *0*	
*Under conditions B4 and B5 the conditional mean difference of each trial is equal to the conditional causal effect of the target population:* *E*[*Y*^*t*^ – *Y*^*t* ′^∣ **X**; *S* = 1] = *E*[*Y*^*t*^ – *Y*^*t* ′^∣ **X**; *S* = m] = *E*[*Y*^*t*^ – *Y*^*t* ′^∣ **X**; *S* = 0]	
*Under conditions B1–B3, the common conditional mean difference is giver from the formula:* *τ*(*t; t ′;* **X**) ≡ *E*[*Y* ∣ **X**; *S* = 1*; T* = *t*] – *E*[*Y* ∣ **X**; *S* = 1*; T* = *t′*] = … = *E*[*Y* ∣ **X**; *S* = m*; T* = *t*] – *E*[*Y* ∣ **X**; *S* = m*; T* = *t ′*]	
*Finally, the ATE for the target population is:* *E*[*Y*^*t*^ – *Y*^*t* ′^∣ **X**; *S* = 0] ≡ *E*[*τ*(*t; t ′;* **X***)* ∣ *S* = 0]	

#### Notation

Let *t* denote the treatment with *t* ∈ ***T*** = (1,…, L) where ***T*** is a finite set of treatments. Let *s* denote the trial with *s* ∈ ***S*** = (1,…, m) where ***S*** is a finite set of trials. Let also *S* = 0 denote the non-randomized target population. Let **X** be a set of observed baseline covariates. E[*Y*^*t*^ − *Y*^*t*’^∣ *S* = 0] denotes ATE in the target population for the treatments *t* and *t’*.

B1 implies that the treatment effect is consistent irrespective of trial participation. Conditions B2 and B3 are expected to hold within trials due to randomization. B4 implies that there is no trial effect affecting ATE conditional on the baseline covariates X. Finally, B5 implies that the probability of observing covariate patterns based on which B4 stands, should be non-zero. Under B1–B5, inferences are transportable from each trial to the target population. Specifically, under B4 and B5, the ATE is independent of study participation in ***S***, within strata of baseline covariates. When multiple trials are pooled, the positivity assumption B5 can be relaxed assuming that trial-specific conditional ATE is equal to the conditional ATE of the target population under a subset of baseline covariates **X** and that the probability of covariate patters occurring under different trials is non-zero.

All conditions B1–B5 assume that there is perfect adherence and compliance in all trials and there is no trial attrition. The authors also provided extensions to the conditions in Table 2 under specific setting where this assumption does not hold.

Based on the above set of identification conditions, Dahabreh et al. [7, 8] provided two estimation approaches. The first approach models the conditional ATE directly from the pool of the trials and baseline covariates. The second estimation method is based on a weighting estimator, that is, applying trial-specific weight based on the probability of trial participation and treatment (see [7, 8] for details). In both cases, the authors suggest that Wald-type or bootstrap-based confidence intervals can be derived. The authors provided code in R for implementation of the above estimands.

### Causal inference for network meta-analysis using summary data from independent RCTs

Schnitzer et al. [9] described a framework where causal estimates can be derived in a network meta-analysis setting by using aggregate data from multiple RCTs. This approach focuses on estimating an average treatment effect under the presence of heterogeneity rising from differences in study-level characteristics. The authors define a marginal and model-independent causal estimand and outline the key assumptions that are required for this estimand to be identifiable under measured study–level confounding. An arm-based network meta-analysis approach is adopted throughout the paper which estimates the arm-specific effects, in contrast to the study-based approach that estimates the study-specific effects.

In the arm-based approach, the authors assume that each trial samples randomly from their respective population and that, due to randomization, each trial arm is representative of their population. Then, the authors define their superpopulation (coining the term *metapopulation*) as the union of the trials’ populations. Due to differences across trial, the authors assume that each trial may not be representative of the superpopulation and that each trial estimates its own effect. Therefore, in order to account for differences across trials, one would have to adjust for variables that contribute to differential treatment selection and to the outcome distribution.

Regarding the computational part, in total, three estimation methods (G–computation, inverse probability of treatment weighting (IPTW), and targeted minimum loss–based estimation (TMLE)) are presented and compared in a simulation study. Briefly, in the G–computation method, a maximum likelihood substitution estimator of the G–formula [10], the authors start by fitting a regression model that estimates the outcome of each arm i in study j using all arm regardless of treatment assignment. In the next step, the predicted study effect under each treatment is estimated and a mean effect across studies is derived. Finally, the standard error of the G–computation estimate is derived by bootstrap methods. The disadvantage of this approach is that the correct model specification is challenging. The second method is based on IPTW where a propensity score that estimates the probability of each arm receiving the treatment is computed and this probability is used as weights to create a “pseudo–population” of study arms that are free from confounding bias from the study-level confounders. The disadvantage of this approach is that the number of study arms must be sufficiently large. IPTW using propensity scores is also subject to correct model specification. The TMLE is a doubly robust method that involves the estimation of arm-based effects by fitting a model for the expected value of the arm-based means and by obtaining the predictions of study-specific effects under treatment. In the next step, these predictions are updated by a no-intercept logistic regression using only the arms under treatment and a single covariate corresponding to the estimated probability form the IPTW method. Under a correct model specification for the propensity score, this method provides a consistent estimate. Based on a simulation study, under a correctly specified model, the G–computation method had the performance followed by the TMLE. G–computation and TMLE methods where more sensitive to model misspecification than IPTW; however, the latter was mode biased and had a larger variance when the number of studies was small.

### Conceptual framework considerations

#### Data generation process and sampling for common, fixed, and random effects

As we already showed, approaches to identification of causal effects under a meta–analytical framework, although scarce, do exist in the literature. However, there exists a more fundamental problem that seems to not have attracted enough attention. This problem pertains to the actual scientific relevance and/or clinical applicability of an otherwise valid causal estimate. This problem directly translates to the specification of the data generation process of the study and participants.

In the medical literature, meta-analysis has been a useful tool for summarizing the plethora of evidence in any specific topic. These two prevalent approaches in undertaking a meta-analysis are commonly known as the fixed-effect and the random-effects models. What is usually overlooked is that there exist in fact two distinct sets of assumptions that lead to the same estimator derived from a fixed-effect model [11] which are denoted as the common effect model and the fixed-effects model. The common-effect model is well known in the literature; it is the “classic” fixed-effect meta-analysis model. In this model we assume that all identified studies are trying to estimate one common-effect *θ* and that all differences between studies are attributed exclusively to the sampling error. The fundamental assumption of this model is that all studies use data from their populations who in turn are random samples from the same superpopulation. Therefore, under the common effect assumption, the estimator $\hat{\theta}$ represents the weighted average estimate of *θ* from the several studies. The exact same estimator however can arise from an entirely different set of assumptions, denoted here as the fixed-effect model. Under this particular model, much like as in the random-effects model, each study’s effect is an estimate of its own *θ*_*i*_. The difference from the random-effects model is that under the fixed-effects model, we assume that *θ*_*i*_s are unrelated. This model merely states that each study estimates its own effect irrespective from the other study effect. In contrast, under the random-effects model, we assume that all study effects are a sample from the distribution of study effects. This means that each study’s effect, albeit different from the other studies’ effects, rises from the same distribution of effects, governed by parameters or characteristics of the mixture of distributions. The difference from common/fixed-effects is that by using random-effects, we shift the focus from describing the intervention effect on the underlying superpopulation to describing the characteristics of the distribution of the effect sizes. These three meta-analytical approaches in fact assume three distinct data generation processes and we argue that, depending on which approach one assumes, the pooled estimate for the causal treatment effect may be biased.

#### Interpretation and scientific relevance of the identified causal effects

Sobel et al. [6] made no remark on the choice of trials included in their meta-analysis. However, based on A7, they consider that each trial samples from its distinct population, which implies that the superpopulation is a mixture of each trial’s population. Schnitzer et al. [9] explicitly stated that the subjects in each trial are assumed to be random samples of their own populations and furthermore define their superpopulation (metapopulation) as the union of each trial’s population. Essentially, these two methodologies assumed that each trial includes a random sample of their specific population and implicitly or explicitly assume that the underlying population of all trials in the union of the trial-specific populations. These descriptions of the superpopulations are in line with the fixed-effects and the random-effects meta-analysis models but not with the common effect model. This does not necessarily translate to the methodologies being completely incompatible with the common-effect model. For example, in Sobel et al. (assuming that the assumptions **A1**, **A2**, and **A6** hold), in the special case where assumptions **A3a** and **A5a** hold, as well as all studies sample randomly from the sample superpopulation (i.e., the assumption **A7** hold unconditionally of the **X**_**2**_) and then it essentially collapses to the common-effect model, irrespective of the model of the effect size *H* specified in the assumption **A4**. A more realistic scenario, however, is when the assumption **A7** holds conditionally, as described in the original paper, where the sample populations are not necessarily equivalent to the superpopulation. Since the set of covariates **X**_**2**_ may include both individual- and study-level covariates, then it seems even more unlikely for **A7** to hold unconditionally, since this would mean at least equivalence of the distributions of the individual-level covariates across studies. On the contrary, the approach of Dahabreh et al. [7, 8] explicitly assumed that the data were obtained by random sampling from an infinite superpopulation (stratified by study *S*). Dahabreh et al. noted that this procedure would lead to a “biased” sample, i.e., the probability of individuals being included in a study differs between the infinite superpopulation and the sampled data, but stated that the identicality of the causal effects is unaffected. The authors acknowledge such a hypothetical “infinite superpopulation” is (similar to every frequentist approach for statistical inference) more of a convenience than a likely existing population of individuals. The same holds for the superpopulation/metapopulation defined in the other methods. Even then, such a superpopulation of *individuals*, seems more plausible compared to an infinite population of *effect sizes* from which the observed study effects are sampled from, under the random-effects model [7, 8, 12]. Not only is it unlikely that such a population of effect sizes exist, but also the interpretation of the summary effect pertains more to the characterization of the said distribution that to the description of the effect on the superpopulation.

Sobel at al. did not explicitly address how their approach corresponds to either common, fixed, or random effects. Instead, the model to be used for the estimation of the effect size was represented by a generalized function *H*. Depending on the statistical model described by the function *H*, all three meta-analysis models can be applied. In the provided example, the model used is similar to a one-stage IPD meta-analysis and equivalent to the common and fixed-effects models. Schnitzer et al. also did not make any explicit assumptions regarding the compatibility of the proposed approach with the three meta-analysis models. However, the simulation study that they performed to compare the efficacy of the estimators only focused on random-effects methods. Finally, the focus of the methodological approach described by Dahabreh et al. is the identification and estimation of a valid causal effect which can be applicable to a specific target population with specific characteristics. Therefore, it is not directly equivalent to any of the three usual meta-analysis models whose primary focus leans towards the generalizability rather that the transportability of the effects.

All methodologies provide a valid approach to estimate an effect that has a causal interpretation in the respective superpopulations. However, it is also important to consider whether these superpopulations are actually plausible populations that exist naturally. In that end, one has to consider the data generation process of the trials. While it is often reasonable to assume that each trial samples randomly from their respective populations, we have to acknowledge that in most cases it seems implausible that these same trials are a random sample of a population of trials. Had we had a random sample of trials, then this sample would allow for the covariate distribution of the superpopulation to be consistent with the covariate distribution of a naturally existing population. However, this is rarely the case, as conducting a trial is largely a function of very specific motives and aims [13, 14], and one may argue that a random sample of trials may not naturally occur. And while this amalgamation of trials does not directly affect the underlying assumptions invoked by a fixed-effects or a random effect meta-analysis, which pertain to the sampling of the *effect estimates* rather than the *sampling of trials* [13, 15], we argue that, without taking into account any potential differences between the structure of the superpopulation and that of a naturally occurring population, the generalizability of the produced results (which would have a pertinent causal interpretation for the superpopulation) would be hindered. Therefore, one must be very careful regarding the generalizability of the meta-analytical causal estimates. One approach would be to ignore this by implicitly or explicitly assuming that the meta-analytical causal estimate for superpopulation is the same or sufficiently close to the actual estimate for the natural population. Alternatively, one could acknowledge this caveat of the generalizability but describe the estimate nonetheless. The obtained estimate would still be the best available description of the effect. The approach of Dahabreh et al. partially ameliorates this by following an alternative approach which focuses on assumption for trial populations rather that assumptions of the trial effects, as discussed earlier.

## Discussion

In this work, we reviewed published methodologies pertaining to causally explicit description of meta-analyses of RCTs and other similar evidence synthesis frameworks, such as multilevel or hierarchical frameworks with regard to obtaining a causally interpretable meta-analysis estimate. Overall, we identified three methodologies directly pertinent to a causally explicit description of a meta-analytical estimate.

The first methodology [6] provided a set of 7 causal inference assumptions under which a meta-analytical causal effect is identifiable and estimable. This methodology required individual participant data from all trials to work, similar to a one-stage meta-analysis. However, this methodology differs computationally from a “classic” one-stage meta-analysis in that it only fits a regression model in contrast to the one-stage meta-analysis where it is standard to use a hierarchical model for estimation. The second methodology [7, 8] was also based on individual participant data from trials and baseline data from a target population using a well-defined causal inference framework. A drawback of this approach is that it requires baseline data from the target population on which the causal effects are to be transported. As it is often unlikely for data from the target population to be readily available, the actual applicability of this approach seems somewhat limited. Finally, the last methodology [9] focused on summary data from network meta-analysis. This approach tries to account for differences across trials in order to estimate a marginal causal effect that refers to a superpopulation defined as the union of the populations the trials sample from. Overall, the methodologies presented in this review address two distinct aspects of statistical inference, the generalizability of the effects in the population from which the data are sampled from and the transportability of the effects, which allows for inferences to a new target population. While both are equally important, our review focused more on generalizability, which addresses the internal validity of the estimates and is more often the focus in the literature of meta-analyses of RCTs.

An inherent limitation of such approaches is the data generation process for the trial selection. Although all methodologies provide valid causal estimates, these are restricted to their superpopulations respectively. As it is rarely the case that the actual trials are random samples from a population of trials that in turn sample randomly form a naturally occurring population, it is evident that these superpopulations may differ substantially from a naturally occurring population. While this problem may not hamper the interpretation of the results from a “classic” meta-analysis where no causal interpretation is made, that is, the description of the distribution of the effect estimates (for random-effects meta-analysis), it would severely hinder any efforts for a causal interpretation of the said results. Therefore, although helpful in summarizing and providing the best description available for this evidence, caution is needed when trying to make inferences. One would have to consider the differences in the structure between the two (naturally occurring and theoretically constructed) superpopulations and consider whether the estimated causal effect is relevant. Only the approaches by Dahabreh et al. [7, 8] recognized this limitation in the data generation process and provided a limited solution under certain assumptions.

There is an extensive literature on the extensions of causal inference focusing on complex single-trial settings [16, 17] on surrogate endpoints [18], or on non-randomized data (observational or quasi-experimental settings) using multilevel or hierarchical frameworks [19]. A comprehensive review of multi-level models for causal inference focusing on randomized experiments in education was recently published [20]. Finally, efforts to synthesize data from multiple sources (observational and experimental) also exist [21]. However, to our knowledge, this is the first systematic effort to survey relevant methodologies which explicitly expand the causal inference framework to incorporate data from multiple trials by using an evidence synthesis approach.

Other approaches exist in evidence synthesis that can be used to investigate causality. A causal inference approach in meta-analysis of RCTs focusing on a slightly different scientific question to what was presented in our review was addressed in Zhou et al. [22] who focused on the estimation of the complier average causal effect (CACE) based on meta-analysis of RCTs with non-compliance. Under the principal stratification framework [23] which takes into account the non-compliance in trials, CACE is an alternative to ATE for the estimation of causal effects. Zhou et al. [22] presented a novel approach in estimating CACE based on a Bayesian hierarchical model by taking into account the study-specific random-effects to account for heterogeneity across trials. Although this study provided insights to the CACE estimation from meta-analysis, it did not provide an explicit causal inference framework, but only reflected upon the single-trial assumptions of the principal stratification framework, leaving aside key components, such as the exchangeability across trials. Mendelian randomization studies are also alternative approaches based on evidence synthesis which can be used to investigate causality [24]. However, the key difference of these methodologies and the ones presented in this paper is that in Mendelian randomization studies, one starts based on the key assumption that the studied association is causal and then proceeds to synthesize the available data. In contrast, causal inference approaches, such the ones presented in this paper, aim to identify potential causal relationships in an observed association.

## Conclusions

Despite both causal inference methodology and meta-analysis of randomized controlled trials being regarded as two of the most useful tools in refining evidence-based hierarchy, there is only limited effort in the bibliography to combine these approaches in order to attain higher levels of causally interpretable evidence. To date, only a limited number of methodological frameworks have addressed this issue, providing ways to obtain causal estimates from meta-analyses of randomized controlled trials. And while all three identified methodologies would produce a valid causal estimate, due to potential violations of study protocols and limitations in the assumptions regarding the data generation process of the potentially included in the meta-analysis RCTs, the interpretation and generalizability of the causal estimands may prove challenging.

## Data Availability

Data sharing is not applicable to this article as no datasets were generated or analyzed during the current study.

## Abbreviations

ATE: Average treatment effect
CACE: Complier-average causal effect
ITE: Individual treatment effect
RCT: Randomized controlled trial
IPD: Individual participant data
IPTW: Inverse probability of treatment weighting
SUTVA: Stable unit treatment value assumption
TMLE: Targeted minimum loss-based estimation
